# *In silico*, biologically-inspired modelling of genomic variation generation in surface proteins of *Trypanosoma cruzi*

**DOI:** 10.1186/1475-9292-6-6

**Published:** 2007-07-10

**Authors:** Francisco J Azuaje, Jose L Ramirez, Jose F Da Silveira

**Affiliations:** 1 Computer Science Research Institute and School of Computing and Mathematics, University of Ulster, Jordanstown, BT37 OQB, Northern Ireland, UK; 2 Biotechnology Centre, Instituto de Estudios Avanzados (IDEA)-MCT, Caracas, Venezuela; 3 Escola Paulista de Medicina, Universidade Federal de São Paulo, Brazil

## Abstract

**Background:**

Protozoan parasites improve the likelihood of invading or adapting to the host through their capacity to present a large repertoire of surface molecules. The understanding of the mechanisms underlying the generation of antigenic diversity is crucial to aid in the development of therapies and the study of evolution. Despite advances driven by molecular biology and genomics, there is a need to gain a deeper understanding of key properties that may facilitate variation generation, models for explaining the role of genomic re-arrangements and the characterisation of surface protein families on the basis of their capacity to generate variation. Computer models may be implemented to explore, visualise and estimate the variation generation capacity of gene families in a dynamic fashion. In this paper we report the dynamic simulation of genomic variation using real *T. cruzi *coding sequences as inputs to a computational simulation system. The effects of random, multiple-point mutations and gene conversions on genomic variation generation were quantitatively estimated and visualised. Simulations were also implemented to investigate the potential role of pseudogenes as a source of antigenic variation in *T. cruzi*.

**Results:**

Computational models of variation generation were applied to real coding sequences from surface proteins in *T. cruzi*: trans-sialidase-like proteins and putative surface protein dispersed gene family-1. In the simulations the sequences self-replicated, mutated and re-arranged during thousands of generations. Simulations were implemented for different mutation rates to estimate the relative robustness of the protein families in the face of DNA multiple-point mutations and sequence re-arrangements. The gene super-families and families showed distinguishing evolutionary responses, which may be used to characterise them on the basis of their capacity to generate variability. The simulations showed that sequences from *T. cruzi *nuclear genes tend to be relatively more robust against random, multiple-point mutations than those obtained from surface protein genes. Simulations also showed that a gene conversion model may act as an effective variation generation mechanism. Differential variation responses can be used to characterise the sequence groups under study. For example, unlike other families, sequences from the DGF1 family have the capacity to maximise variation at the amino acid level under relatively low mutation rates and through gene conversion. However, in relation to the other protein families, they exhibit more robust behaviour in response to more severe modifications through intra-family genomic sequence exchange. Independent simulations indicate that DGF1 pseudogenes might play a role in the generation of greater genomic variation in the DFG1 gene family through gene conversion under different experimental conditions.

**Conclusion:**

Digital, dynamic simulations may be implemented to characterise gene families on the basis of their capacity to generate variation in the face of genomic perturbations. Such simulations may be useful to explore antigenic variation mechanisms and hypotheses about robustness at the genomic level. This investigation illustrated how sequences derived from surface protein genes and computer simulations can be used to investigate variation generation mechanisms. Such *in silico *experiments of self-replicating sequences undergoing random mutations and genomic re-arrangements can offer insights into the diversity generation potential of the genes under study. Biologically-inspired simulations may support the study of genomic variation mechanisms in pathogens whose genomes have been recently sequenced.

## Background

*Trypanosoma cruzi *is the etiological agent of Chagas disease, which is an incurable and debilitating illness affecting millions of people in Latin America [1,2]. Chagas disease also represents a serious health concern for industrially-developed countries. There is a potential for infection in the USA and Europe due to the risk of contamination of the blood supplies. Additionally, HIV/AIDS patients may experience reactivation of Chagas disease [3]. *T. cruzi *infects and adapts to the vertebrate host by exploiting evolutionary strategies to invade target cells and to evade (or to confuse) the immune system [4,5]. The invasion, evasion and infection process involve different families of surface proteins [5]. The generation and presentation of variable surface antigens is a key strategy [5-7]. The parasite may take advantage of this strategy to adhere to different molecules on the host cell membrane and the extracellular matrix [5].

In general, an understanding of the roles of genetic damage (mutations) and recombination in the generation of antigenic diversity in trypanosomes is important for addressing key questions about the evolution, adaptation and robustness of parasites and their interactions with the hosts. However, the experimental analysis of antigenic diversity generation represents a massive challenge, even in the case of parasites that have traditionally received relatively more international attention than *T. cruzi*, such as the malaria and sleeping sickness parasites. The availability of gene sequences derived from the *T. cruzi *Genome Project [8] further motivates the proposal of data- and discovery-driven approaches to characterising functional properties of proteins at different levels of organisation.

The computer-based, dynamic modelling of biological processes and mechanisms may provide tools to aid researchers in addressing fundamental questions about the genomic basis of evolution, adaptation and complexity. The area of *digital genetics*, also known as *artificial life*, offers tools in which self-replicating strands of computer code are capable to mutate, compete, evolve and adapt to a computing environment with space and resource constraints. Darwinian evolution has also inspired the development of the area of *evolutionary computation*, which provides algorithms that mimic mutation, recombination and selection mechanism of data structures based on predetermined "fitness" functions. Evolutionary computation methods, such as *genetic algorithms*, have been successfully applied to solve optimisation problems in many disciplines. The reader is referred to [9] and [10] for reviews of the areas of digital genetics and evolutionary computation.

In a previous work [11] we investigated the potential capacity of *T. cruzi *surface protein genes to maximise phenotypic variation, which may be seen as a key attribute to expand the repertoire of surface antigens. The robustness of a parasite gene against mutations was addressed in terms of several gene *volatility *and *diversity *indicators. The potential impact of point-mutation errors on surface antigen genes based on the analysis of codon usage and its potential for generating different amino acid mutants were explored.

In this paper we propose *in silico*, yet biologically-inspired, models that dynamically simulate genomic variation generation through random mutations and a relatively simple approximation of recombination. The models and simulations implemented in this exploratory study processed coding sequences from two surface protein super-families: the *TS (trans-sialidase)-like proteins *and *putative surface protein DGF-1 *(dispersed gene family-1) super-families [5,8,12]. These families are known to be important components in the infection process, but relatively little is known about their functional and adaptive properties. Moreover, the mechanisms underlying their variation generation process are not well-understood.

We propose the implementation of dynamic simulations of mutation and genomic arrangements to estimate the antigenic generation capacity of these families. In this approach sets of gene sequences of biological interest for the study of *T. cruzi *are used as inputs to a system that recreates mutation and replication mechanisms. The system can also be used to simulate a gene conversion mechanism under different *in silico *conditions, such as *mutation rates *and the number of *simulation generations*. Such simulations allowed us to visualise the generation of variation at the amino acid level using the available sequences (inputs) as starting points, as well as comparative references, in the simulations. Thus, this methodology has the potential to be applied for assessing the effect of mutation and genomic exchange (e.g. gene conversions) in the generation of diversity of surface proteins. Moreover, it may be exploited to characterise gene families in terms of putative models of antigenic variation through mutation and conversion. This investigation explores some of these applications and discusses their potential implications for addressing fundamental questions relevant to the understanding of mechanisms driving genomic diversity.

## Results

A variation generation simulation require DNA coding sequences, a user-defined mutation rate value and the number of simulation steps (self-replication generations) as inputs. The input sequences are used as variation references, i.e. the degree of variation of subsequent mutated sequences is measured in relation to these input (wild) sequences. *The variation *of a given sequence at a particular time is estimated here by measuring the divergence (distance) between the resulting amino acid sequence (encoded by a mutated DNA coding sequence) and the reference amino acid sequence. Mutations, i.e. random multiple-point mutation and sequence re-arrangements, occur at the DNA level. At each generation step a multiple-point mutation or a gene conversion event occurs, depending of the model being simulated. The degree of variation for each gene sequence is calculated based on the resulting amino acid sequence. An *average variation *value for each family of genes is calculated at each simulation step. At the end of a simulation the system outputs the average variation values at each simulation step for the sequence family under consideration. The section of Methods provides more details about these information processing steps.

Different simulations using genes from the TS and DGF1 superfamilies were implemented. The TS family consists of a large number of genes that encode major surface antigens of the infective forms of *T. cruzi *[5]. This super-family can be categorised into four groups according to sequence similarity, molecular mass and function [5,8]. There are 1430 TS sequences in the *T. cruzi genome*: 737 genes and 693 pseudogenes [8]. In this investigation we concentrated on 261 complete TS proteins whose biological and structural properties had been previously described. The TS super-family is divided into 14 families [5,11]: ASP-1 (25 sequences), ASP-2 (37 sequences), CEA (17 sequences), CRP-10 (24 sequences), FL-160 (16 sequences), GP82 (19 sequences), GP85 (92 sequences), MVar1-GP90 (101 sequences), SA-85 (98 sequences), SAPA (30 sequences), Tc85-11 (93 sequences), TESA-1 (57 sequences), TS EPI (30 sequences) and TSA (37 sequences). The DFG1 genes [12,13] investigated here were represented by 85 coding sequences.

It has been suggested that genes encoding proteins involved in maintaining core biological functions, such as house-keeping and nuclear proteins, tend to be genetically robust to mutations (at the protein level). Therefore, simulations were also performed on a set of non-surface sequences to illustrate possible differences between genes from these cellular components. We selected a group of 40 sequences encoding nuclear proteins in *T. cruzi *to further illustrate observed differences between these families and as a baseline reference for discussions. The potential role of pseudogenes in the generation of genetic variability was also explored by performing independent experiments in which 15 DGF1 pseudogenes and 23 GP85 pseudogenes from the group II of TS superfamily drove random genomic modifications in their respective gene families. Thus, pseudogene sub-sequences provided the potential sources of variation in random gene conversion events. The resulting variation patterns were compared to those obtained from simulations that did not involve the participation of pseudogenes.

### A multiple-point mutation model

**Figure 1:**
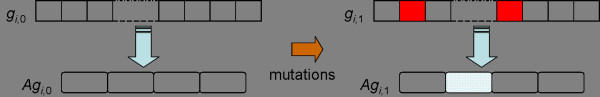
Multiple-point mutation model. A (reference) input DNA sequence, *g*_*i*,*0*_, encodes an amino acid sequence, *Ag*_*i*,*0*_, at generation '0'. Random selection and mutation of nucleotides generate a new sequence, *g*_*i*,*1*_, at generation '1', which encodes a mutated amino acid sequence, *Ag*_*i*,*1*_. The variability of a gene *i*, *g*_*i*,*j*_, at generation *j*, is estimated by measuring the difference between *Ag*_*i*,*0 *_and *Ag*_*i*,*j*_.

This first variation generation model evaluated simulates random multiple-point mutations at the nucleotide level. The amount of mutations occurring in a sequence depends of a *mutation rate *(*mr*), which is directly proportional to the percentage of nucleotides to be mutated in a specific gene sequence. A mr = 0.0005, for example, means that at each simulation step (generation) 0.05% (0.0005 × 100%) of the nucleotides will be randomly selected and mutated. Each nucleotide in the (mutating) sequence has the same probability of being selected. Also each nucleotide base has the same probability of being chosen to substitute a base in a mutating sequence. Gene sequences are replicated and mutations are accumulated from generation to generation. Figure 1 illustrates this variation generation model. At each generation the resulting (mutated) amino acid sequence is compared to the reference (input) amino acid sequence and their distance in the sequence space is calculated. Such a distance is used to estimate the variation or diversity generation capacity of a gene (more details in Methods). Simulations were performed on each family and distance values were calculated for each sequence at each simulation step (i.e. generation). Mean distance values were used to characterise the (average) variability of a family for a specific mr value at each simulation generation. The results reported in this paper were obtained from simulations obtained with 1000 generations (replication-mutation steps).

**Figure 2:**
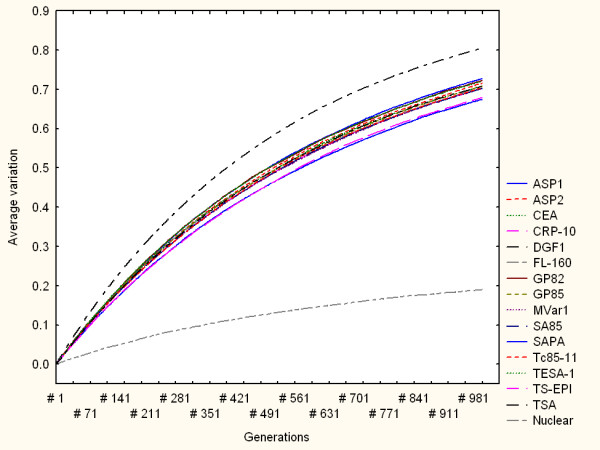
Simulation results from a multiple-point mutation model for all the families when mr = 0.001. The horizontal axis indicates the simulation steps (generations). The vertical axis shows the average variation of the protein family measured as the average distance from the mutated amino acid sequences, at a particular time (generation number), to their respective reference sequences. Each curve refers to the average variation response of a family to multiple-point mutations. Top and bottom curves represent the average variation of DGF1 and nuclear amino acid sequences respectively.

**Figure 3:**
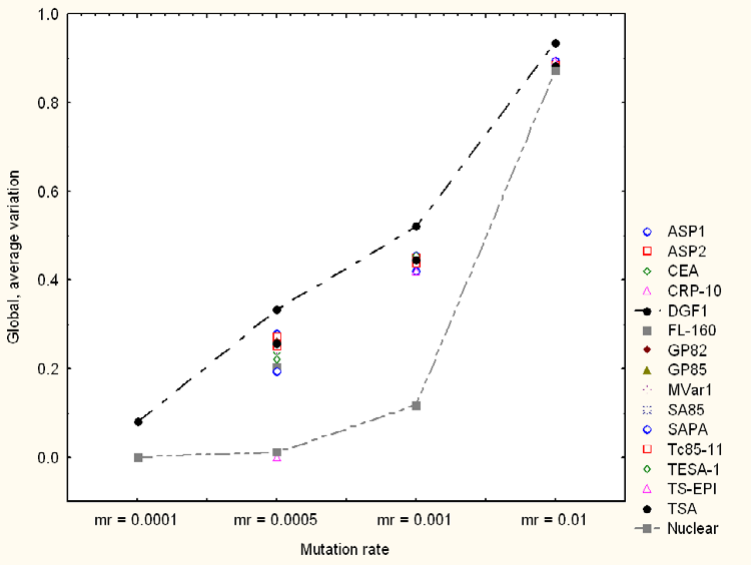
Variation model based on multiple-point mutations. Summary of global variation generation under different mr values. Each point represents the global, average variation values observed in independent simulations for each protein family through 1000 generations. Upper and lower curves highlight the global, average variation of DGF1 and nuclear amino acid sequences respectively.

Figure 2 shows the simulation results for all the families when mr = 0.001. In this and similar figures the horizontal axis indicates the simulation steps (generations) from 1 to 1000 generations. The vertical axis shows the average variability of the protein family measured as the average distance from the mutated amino acid sequences, at a particular time (generation number), to their respective reference amino acid sequences. Each curve refers to the average variation response of a family to multiple-point mutations. The closer a curve is to the top of the graph the higher the average amino acid variability observed. This simulation, as well as other with different mr values, consistently shows a relatively higher variation capacity of DGF1 in comparison to the other surface genes considered here. As expected, genes encoding nuclear proteins tend to be, in general, more consistently robust to random mutations than surface genes. Some surface gene families display more heterogeneous responses. For example, TS-EPI may exhibit a relative more robust response to multiple-point nucleotide sequences (lower diversity generation) for mr below 0.0005. Figure 3 summarises the genomic variation generation responses of all the families under different mr values. Note that for mr = 0.0001 only DGF1 generated variable amino acid sequences. In this figure only the global, average variation values from the families with the highest (DGF1) and lowest (nuclear) diversity generation capacities are linked with lines.

### A variation generation model based on gene conversion

The second variation generation model explored mimics genomic exchange through recombination. This model may be related to a gene conversion model based on synthesis dependent strand annealing (SDSA) homologous recombination [14]. Since unequal recombination mechanisms involving chromatids exchange at chromosomal interstitial areas may lead to alteration in syntheny, which is not the case in trypanosomatids so far studied[15], we envisioned that the recombination mechanisms operating in this exchange can be defined as gene conversion vía SDSA homologous recombination [14] without crossover.

**Figure 4:**
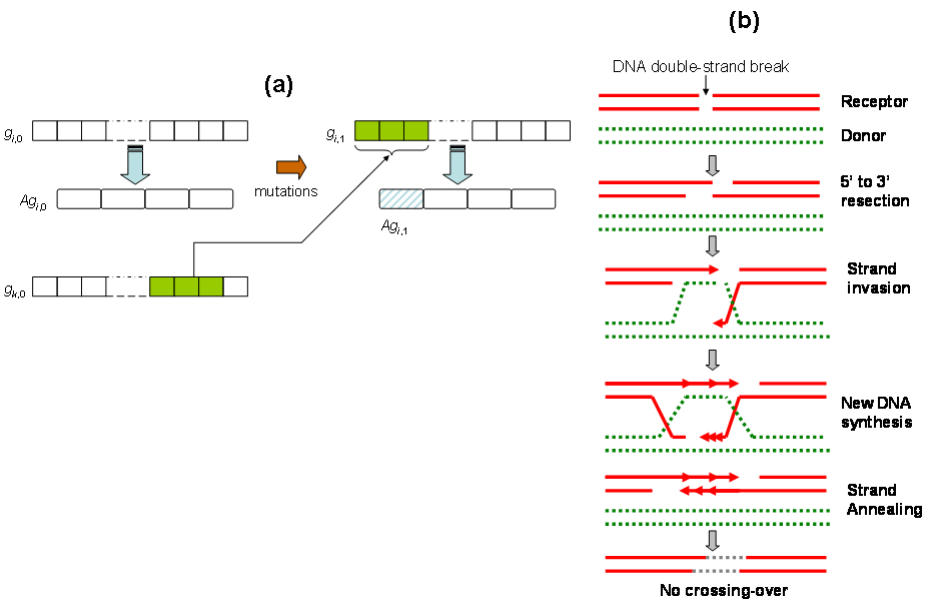
A variation generation model based on gene conversion. (a) At generation '0', a (reference) native DNA sequence, *g*_*i*,*0*_, encodes a amino acid sequence, *Ag*_*i*,*0*_. The mutation of nucleotides generate a new sequence, *g*_*i*,*1*_, at generation '1', which encodes a mutated amino acid sequence, *Ag*_*i*,*1*_. This is achieved when a donor sequence, *g*_*k*,*0*_, is randomly selected to provide a sub-sequence to *g*_*i*,*0 *_. The resulting mutated sequence at generation '0' encodes *Ag*_*i*,*1*_. The variability of a gene *i*, *g*_*i*,*j*_, at generation *j*, is estimated by measuring the difference between *Ag*_*i*,*0 *_and *Ag*_*i*,*j *_. (b) A more detailed graphical illustration (at the DNA level) of the biological gene conversion model based on SDSA homologous recombination (adapted from [14]), which is computationally approximated by model summarised in (a).

In the computational model implemented here gene sequences from the same family randomly exchange nucleotide sub-sequences, which may encode diverse amino acid sequences (Figure 4(a)). Figure 4(b) offers a more detailed graphical illustration (at the DNA level) of the biological gene conversion model based on SDSA homologous recombination, which may be computationally associated with the model summarised in Figure 4(a). At a given simulation step (e.g. generation 0 representing the beginning of the simulation) each of the sequences used as inputs to the simulation system undergoes multiple-point, continuous mutations, whose length is defined by a mutation rate, mr. As in the variation generation model introduced above, mr reflects the percentage of nucleotide bases to be randomly mutated. The starting point of a mutation for a given sequence is randomly selected. This is followed by the random selection of a donor sequence from the same family, which represents the source of mutating nucleotides for the sequence to be re-arranged. The starting point of the sub-sequence to be donated is randomly selected and its length is defined by the mr value. The re-arranged sequence encodes a mutated amino acid sequence, which is compared to the reference or native sequence (original input sequence). As in the multiple-point variation model introduced above, the variation level of a sequence is estimated by calculating the distance between the mutated amino acid sequence (at any generation during the simulation) and the reference sequence (at generation 0). Average variation values are calculated for each family. Results reported here were observed for simulations implemented with different mr values and during 1000 generations.

**Figure 5:**
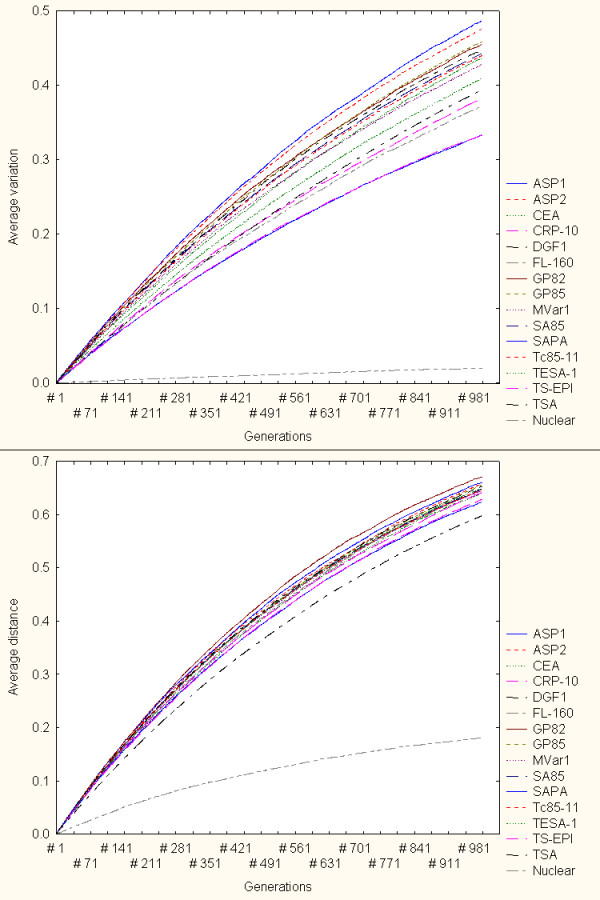
Variation generation through gene conversion. Simulation results with mr = 0.0005 (upper panel) and mr = 0.001 (lower panel). In both panels the lower curves represent the variation generation response of genes encoding nuclear proteins (i.e. most robust family). Under this model the DGF1 becomes relatively more robust to mutations (lower variation generation) for relatively higher mutation rates (lower panel, curve above nuclear genes curve).

Figure 5 summarises the simulation results with mr = 0.0005 (upper panel) and mr = 0.001 (lower panel). In both panels the lower curves represent the variation response of nuclear proteins, which again indicates the relatively low variation generation capacity of this family. Thus, the genes encoding nuclear proteins tend to be genetically robust against these genomic re-arrangements in relation to the surface protein genes. However, for very high mutation rates (mr > 0.01) this family can generate greater diversity (not shown in Figure 5), which may be even comparable to surface genes. In comparison to the other families, the DGF1 family tend to exhibit highly volatile responses to re-arrangements driven by relatively low mutation rates (mr < 0.0005) (not shown in Figure 5). However, the diagrams shown in Figure 5 suggest that DGF1 may exhibit relatively high robust responses (i.e. lower variation generation) in the face of stronger gene conversion-driven perturbations (i.e. mr > 0.0005). The lower panel of Figure 5 indicates that DGF1 genes may generate less genetic diversity (i.e. most robust behaviour) than the other surface protein gene families considered here. In this specific simulation scenario (mr = 0.001) only the group of genes encoding nuclear proteins generated less genetic variability than DGF1 (bottom dotted curve).

**Figure 6:**
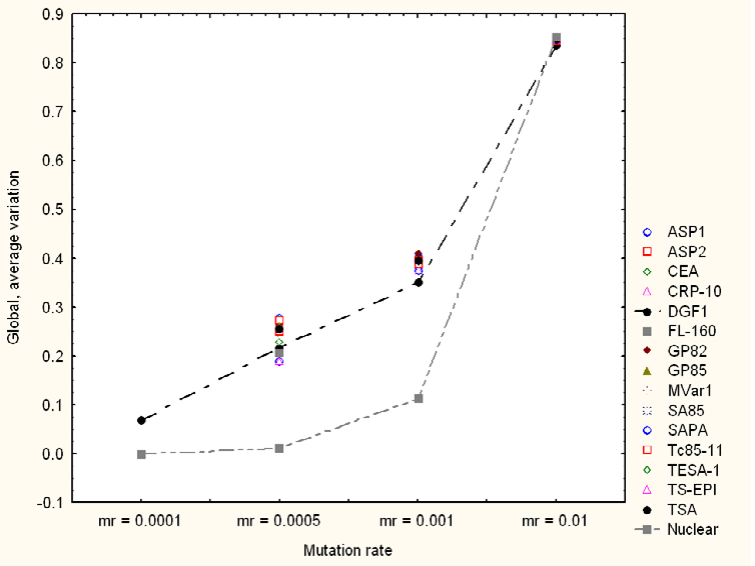
Variation model based on gene conversion. Summary of global variation generation under different mr values. Each point represents the global, average variation values observed in independent simulations for each protein family through 1000 generations. Dotted-line curves highlight the global, average variation of DGF1 and nuclear proteins.

Figure 6 summarises the variation generation patterns of all the gene families for different mr values. In this figure each point represents the global, average variation values observed in independent simulations for each protein family through 1000 generations. Line-dotted curves highlight the global, average variation of DGF1 and nuclear proteins. Note that for mr = 0.0001 DGF1 was the only family that generated variable amino acid sequences. The response of the gene group encoding nuclear proteins is in general consistent with the relative high robustness observed in the multiple-point mutation model simulations (Figure 3). However, under this variation generation model DGF1 exhibits a more robust (less diversity generation) for relatively higher mutation rate values (mr > 0.0005). For mr values greater than 0.0005 the DGF1 represented the most robust surface gene family. Furthermore, for very high mr values (mr > 0.001) DGF1 displayed the lowest diversity generation capacity (highest genetic robustness) in comparison to the other families. The latter two observations are consistent with genetic robustness estimations (based on static volatility scores) reported elsewhere [11].

### An exploration of the potential role of pseudogenes in variation generation

**Figure 7:**
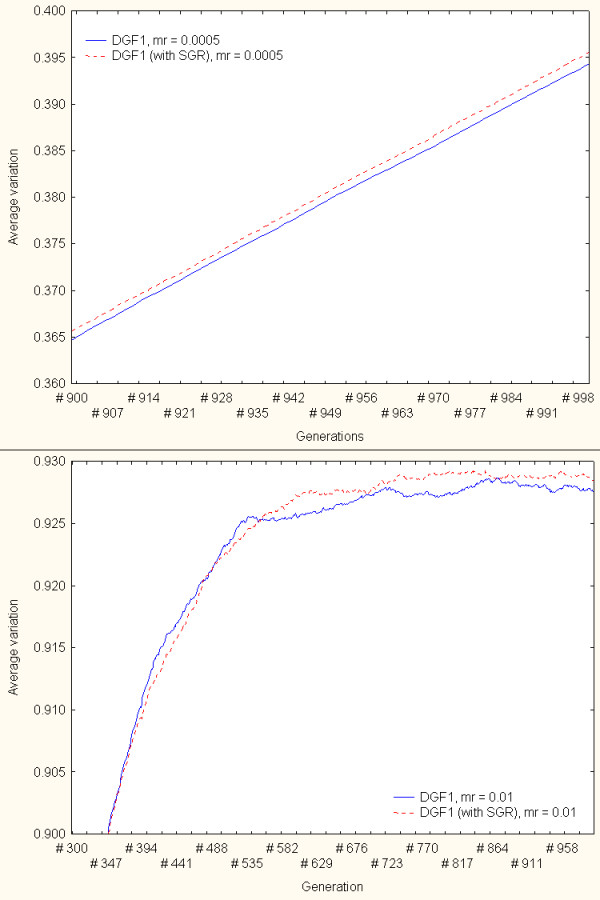
An exploration of the role of pseudogenes in variation generation – DGF1 family. The continuous and dotted-line curves represent the simulation results from variation generation models based on intra-family gene sequence exchange only (pseudogenes were not included in the gene conversion events) and through gene conversion with pseudogenes (with SGR), respectively. The upper panel displays results when the simulations were conducted with mr = 0.0005. The lower panel shows a close up view of results under mr = 0.01.

Further simulations of the gene conversion-based variation generation model were implemented, which first focused on DGF1 genes and pseudogenes. In these independent simulations the DGF1 sequences randomly exchanged sub-sequences with DGF1 pseudogenes only. All pseudogenes had the same probability of being selected to donate a sub-sequence. Moreover, the conversion (insertion) points in the genes (receiver sequences) and pseudogenes (donor sequences) are also randomly selected. Figure 7 depicts different views of the obtained results. The continuous- and dotted-line curves represent the simulation results from variation generation through intra-family gene sequence exchange only (i.e gene conversion in which pseudogenes were not included) and through gene conversion with pseudogenes only, respectively. The upper figure panel displays results when the simulations were conducted with mr = 0.0005. The lower panel shows a partial view of results under mr = 0.01. The results suggest that, for different mutation rates, pseudogenes tend to contribute to the generation of variability. Figure 7, for instance, indicates that gene conversion with pseudogenes may generate greater variation in comparison to the model that does not incorporate peseudogenes. Independently of the mutation rate, there is a time (simulation generation) in which the gene conversion model with pseudogenes will show a higher average variation than the model without pseudogenes. However, the differences between these models do not appear to be statistically significant. These exploratory simulations and analyses suggest that pseudogenes might contribute to the generation of variability in this family. Such a tendency is incrementally reinforced in time, and is maintained after reaching a number of generations (depending of the mutation rate).

**Figure 8:**
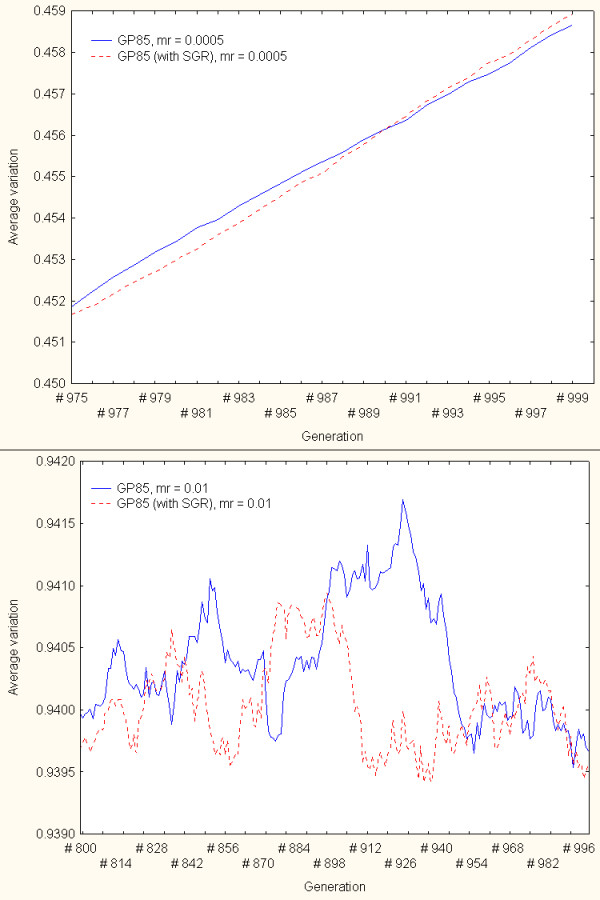
An exploration of the role of pseudogenes in variation generation – GP85 family. The continuous and dotted-line curves represent the simulation results from variation generation models based on intra-family gene sequence exchange only (pseudogenes were not included in the gene conversion events) and through gene conversion with pseudogenes (with SGR), respectively. The upper panel displays results when the simulations were conducted with mr = 0.0005. The lower panels shows a close-up view of results under mr = 0.01.

These simulations were repeated (independently) on the GP85 family only. The results obtained from these simulations showed less clear relationships than in the DGF1 analyses. Unlike in the case of the DGF1 family, GP85 pseudogenes did not achieve a capacity to consistently generate greater variation in comparison to the variation produced by gene conversion without pseudogenes. Figure 8 illustrates different views of some of the simulation results. Its upper figure displays a partial view of results when the simulations were conducted with mr = 0.0005. The lower panels shows a close-up view of results under mr = 0.01. The results from these and other mutation rates indicated that gene conversion with pseudogenes may generate slightly greater variation (in comparison to the model that does not incorporate peseudogenes) during alternating simulation generations only. But unlike the results from the DGF1 family, the capacity of pseudogenes to increase variability through gene conversion is never maintained. Independently of the mutation rate values selected, there are alternating periods in which gene conversion without the participation of pseudogenes may actually contribute to the generation of (insignificant) greater variation.

## Discussion and conclusion

### Potential biological relevance of models and observations

The study of mechanisms for the generation of genetic diversity is important because such mechanisms are needed for the survival and adaptation of the parasite in the hosts. *T. cruzi *exploits such a capacity to generate a massive genetic heterogeneity to increase its chances to adapt to different hosts. The study of genomic variation generation may have applications and implications for the development of novel therapies against these parasites. For example, depending on the level of antigenic variation of a potential vaccine target, different strategies (e.g. focus on function domains, focus on the vector-specific surface antigens) may be considered in the absence of multivalent solutions [16]. Thus, highly volatile (or anti-robust) genes may not be suitable drug targets, in comparison to relatively more robust genes. Relatively more robust genes (i.e. less diversity production ability) might represent an even more feasible target if the gene in question is predicted to be essential for the survival of the pathogen.

Previous research has shown that homologous recombination and gene conversions may be a significant mechanism for achieving antigenic variation [16]. Recent investigations have shown the importance of DNA recombination for the generation of genetic variability in protozoan parasites, including African trypanosomes [17]. However, there is a relative lack of research on genes and mechanisms relevant to DNA mutation, recombination and repair in this and other kinetoplastid organisms.

An important feature illustrated by the proposed methodology is the differentiating response capacity of specific gene families in the face of random mutations. Previous research has suggested that mutation rates (and its effects) strongly depend on an evolutionary compromise between a need to create diversity (a basis for adaptive evolution as in the case of surface protein genes) and a need to preserve core or essential cellular functions (as in the case of genes encoding nuclear proteins) [17]. The results obtained here underlined such a principle and indicated distinguishing genetic variability responses between the gene families analysed. Moreover, the simulations showed differential patterns and responses between surface gene families, which may be used to implement other sequence-based characterisations.

Although genetic exchange among distantly related strains of *T. cruzi *could occur, asexual reproduction is by far the most prevalent way of propagation in this parasite [18,19]. *T. cruzi *displays high level of genetic diversity in the different isolates and even in the cloned cultures [8,20]. Genomic rearrangement including single nucleotide replacement could play a key role in maintaining genetic heterogeneity of its population. Our results are in agreement with previous experimental results that show the importance of genomic rearrangement in the evolution of *T. cruzi *multigenic families [21]. For instance, the diversity found in *T. cruzi *mucin genes of group I (TcMUC I) are due to some point mutations, but mainly to insertions/deletions of complete codons, whereas in the group II (TcMUC) most of the differences are due to non-synonymous point mutations and some small insertions/deletions [21]. These authors suggested that the accumulation of non-synonymous point mutations could be the mechanism involved in the generation of diversity within *T. cruzi *mucin genes [21].

Previous experimental investigations have suggested that genetic re-arrangements based on recombination might be relevant to DNA damage repair mechanisms [17,22]. Thus, DNA recombination or gene conversions may play a role in the reduction of variability. Although our models did not directly address specific aspects of DNA damage and repair through recombination, our results point out interesting quantitative patterns and indicators. For example, our simulations showed that genomic rearrangements by a gene conversion mechanism tend to produce less variable modifications (at the protein sequence level) in comparison to the diversity generated by pure random, multiple-point mutations. This behaviour was observed, for instance, in the DGF1 family in terms of overall average values of genetic variation. Figures 3 and 6 (as well as other simulation result figures) depict that for the same amount of mutated nucleotides (i.e. the same mutation rates), the DGF1 genes generated less variable amino acid sequences through (intra-family) gene conversion than through pure random, multiple-point mutations.

Our simulations indicated that gene conversion might act as an effective variation generation mechanism in TS family. Our results are in agreement with experimental results [23] on TSA genes (group II of TS superfamily). Comparison of TSA genes revealed that they are analogous, supporting the hypothesis that short segments between the family members are exchanged by gene conversion events [23]. Another critical factor that should be taken into account to interpret the gene conversion simulation results is the genomic diversity that is already present in the gene sequences under study, particularly in TS family. The potential roles of such a rich diversity should be considered in future modelling studies.

The possibility that sequence length may be an influential factor in the simulations is excluded by the following evidence. First, we ensured that the amount of mutating bases in each simulation step is defined in relation to the length of the sequence under consideration. That is, the number of mutations and the variation effects are always estimated relative to each sequence, and not as absolute measures. But more importantly, in a previous research we did not detect quantitative correlations between sequence length and variation potential in each of the gene families analysed here [11].

The simulations implemented here also illustrated how the participation of pseudogenes might contribute to an increase in diversity generation through gene conversion. However, our simulations suggested this potential role in the case of the DFG1 family only. More clear or significant relationships between the participation of pseudogenes and genomic variation generation through gene conversion were not observed in the case of the GP85 family. Therefore, there is a need to implement additional, more powerful statistical tests to identify possible systematic differences. Previous experimental research has provided evidence that combinations of silent genes may be involved in the generation of diverse surface proteins in trypanosomes. This may be achieved based on partial duplications of pseudogenes or the insertion of sub-sequences from the silent donor into the variable gene. Recombinational processes have been proposed as a key mechanism for diversity generation, in which the rearrangement of sequences may allow the partial expression of pseudogenes [24]. For example, in *T. brucei *arrangements based on duplicative transposition of pseudogene subsequences have been suggested as an important source of genetic variation [25]. Moreover, the existence of expressed sequences derived from combinations of several donor pseudogenes has been demonstrated. At least in the case of African trypanosomes such pseudogene-based recombinational processes have been suggested as a key mechanism to enhance antigenic diversity of surface protein gene sequences [25]. Further studies (experimental and bioinformatics using these and other protein families) are required to characterise and explain potential roles of *T. cruzi *pseudogenes in the generation of genetic diversity. For example, the results obtained in the GP85 family analysis might provide a basis for motivating the study of the potential role of pseudogenes in protein family preservation or stability. Future studies should also include additional, more powerful statistical analyses to test for systematic effects.

### Summary of key findings and contributions

The simulations allowed us to assess the relative robustness or variation capacity of the protein families in the face of multiple-point mutations or sequence re-arrangements. Distinguishing evolutionary responses were observed between families, which may be used to characterise them on the basis of their capacity to generate genetic diversity. A group of genes encoding nuclear proteins showed an ability to minimise the phenotypic variation generated by random, multiple-point mutations in comparison to the responses observed in the surface protein genes. Simulations also showed that a gene conversion model can act as an effective variation generation mechanism in the DGF1 family.

This and research published elsewhere [11] suggest that the variability patterns and responses observed may be useful features to characterise gene groups. For example, in relation to the other families, sequences from the DGF1 family have a greater capacity to maximise variation at the amino acid level under relatively low mutation rates and through gene conversion. However, in relation to the TS families, the DGF1 family exhibited more robust behaviour in response to more severe mutations through gene conversion. This investigation also illustrated and evaluated a mechanism of genetic variation generation at the amino acid level that was driven by pseudogenes. Simulations using DGF1 sequences indicated that pseudogenes might contribute to the generation of variation of genes through gene conversion-based re-arrangements under different experimental conditions.

Thus, although this research does not report conclusive and experimentally-validated results, it proposes digital dynamic simulations as a tool to support the characterisation of gene families on the basis of their capacity to generate variability in the face of genomic perturbations. These simulations require minimum user-defined inputs and relatively low mathematical complexity to describe models and outcomes. These and future *in silico *experiments of self-replicating sequences undergoing random mutations and genomic re-arrangements can also offer insights into the mechanisms underlying variation generation of the genes under study.

To the best of our knowledge this is the first computational simulation study involving these variation models, sequence data and the proposed simulation approach. A recent study by Lythgoe *et al*. [26] presents a mathematical model of the ordered appearance of variants in *Trypanosoma brucei *during infection. Such a model is based on sets of differential equations describing the dynamics of the host-parasite responses. In our study the only critical, user-dependent parameters are the mutations rates, which allow the user to perform different simulations. The patterns and responses shown here do not depend on other analytical factors or computing variables. As part of future work we will make a computing platform-independent, user-friendly tool publicly available.

The proposed method may be applied as an alternative approach to characterising surface protein families on the basis of the visualisation and quantitative estimation of genomic variation. Therefore, we aim to extend the proposed methodology in order to approach such modelling challenges in *T. cruzi *and other kinetoplastid organisms. Nevertheless, in the long term the scientific value of these and future *in silico *modelling proposals will greatly depend on the availability of experimentally-obtained data, which should be used to corroborate, modify and refine models and simulations. At the time of completing this revision we have not identified a specific strategy or experimental technique to verify all the results reported here. Regarding a possible approach to confirm the nature of the variability of the TS superfamily, the approach implemented by Khan *et al*. [6] based on monoclonal antibodies is a feasible solution. However, this type of approach can now be assessed through new highthrouput techniques, such as antibody-microarrays, which could tell us how many different TS or DGF-1 members are being expressed at a given time in different *T. cruzi *populations. Moreover, we expect that these and future results will help us to direct our attention to more specific experimental approaches that may allow us to test these or related hypotheses.

## Methods

### The data

In this investigation we concentrated on 261 complete TS proteins [8] whose functional and structural properties had been previously described. The DFG1 genes investigated here were represented by 85 coding sequences [8,12]. The TS super-family consisted of 14 families: ASP-1 (25 sequences), ASP-2 (37 sequences), CEA (17 sequences), CRP-10 (24 sequences), FL-160 (16 sequences), GP82 (19 sequences), GP85 (92 sequences), MVar1-GP90 (101 sequences), SA-85 (98 sequences), SAPA (30 sequences), Tc85-11 (93 sequences), TESA-1 (57 sequences), TS EPI (30 sequences) and TSA (37 sequences) [8,23,27-39]. Also our analyses incorporated 40 sequences encoding nuclear proteins in *T. cruzi *to further illustrate observed differences between these families. This group represented a set of sequences described as "nuclear" proteins in the *T. cruzi *Genome Resource [40]. The variation generation simulation analyses also included 15 DGF1 and 23 GP85 pseudogene groups [8,12,13,39], which represented the donor sources in the intra-family gene conversion model. The sequences used in these analyses were obtained after performing Blastp searches in the GenBank database [41] using sequence probes identified by the authors. The sequences were chosen based on their relationship with genes sequences isolated from different laboratories, and whose functions have been confirmed/demonstrated experimentally. For instance, trans-sialidase genes code for proteins with enzymatic trans-sialidase enzyme activity; GP82 and Tc85 have adhesin/binding properties; CRP has complement activity, etc. In the case of the DGF-1 genes used here, their sequences have been validated by comparisons with our own sequences (2 of them) [13] and the original report by Wincker *et al*. [12]. Since the selection of genes was done considering the entire ORF of 10,000 nucleotides long, and comparing these three sequences and the ones in the database, it is unlikely that we have mosaics or misassembled DGF1 genes sequences. Although we cannot rule out the existence of misassembled sequences, such sequences would represent exceptions in the *T. cruzi *Genome Resource.

### Estimation of gene variation

The implemented models quantitatively estimated genetic variation or diversity by calculating the distance between the amino acid sequence resulting from a mutated gene sequence and the corresponding native amino acid sequence encoded by the input gene sequence. Average and overall average variation values were used to summarise variability responses in each family. Distances were calculated by counting the number of different pair-wise amino acids at the same position in the respective sequences. This distance metric is also known as the Hamming distance between two symbol sequences of equal length. It calculates the number of amino acid positions for which the corresponding amino acids differ. In this study each distance was scaled to the length of the sequence under consideration.

### Computational models

Figures 1 and 4 depict the two variation generation models implemented. A variation generation simulation require DNA coding sequences, a user-defined mutation rate, mr, value and the number of simulation steps (self-replication generations) as inputs. In both models mr is proportional to the percentage of nucleotide bases to be mutated. A mr = 0.0005, for example, means that at each simulation step (generation) 0.05% (0.0005 × 100%) of the nucleotides will be randomly selected and mutated. Each nucleotide in the (mutating) sequence has the same probability of being selected. Each nucleotide base has the same probability of being chosen to substitute a base in the mutating sequence. Gene sequences are replicated and mutations are accumulated from generation to generation. In the gene conversion-based model each of the sequences used as inputs to the simulation system undergoes a multiple-point, continuous mutations, whose length is defined by a mutation rate, mr. The starting point of mutation for a given sequence is randomly selected. This is followed by a random selection of a donor sequence from the same family, which represents the source of mutated nucleotides for the sequence to be re-arranged. The starting point of the sub-sequence to be donated is randomly selected and its length is defined by the mr value. The re-arranged sequence encodes a mutated amino acid sequence, which is compared to the reference sequence (original input sequence) like in the case of the first model. Results reported here were observed in simulations implemented with different mr values and a 1000 generations.

The input sequences (see data description) are used as references to estimate variation, i.e. the degree of variation of subsequent mutated sequences is measured in relation to these input (native) sequences. Mutations, i.e. random multiple-point mutation and sequence re-arrangements, occur at the DNA level. At each generation step a multiple-point mutation or conversion event occurs. Both models were implemented independently. The variation value for each gene sequence is calculated based on the resulting amino acid sequence as explained above. An *average variation *value for each family of genes was calculated at each simulation step. At the end of a simulation the system outputs the average variation values at each simulation step for the sequence family under consideration.

## Competing interests

The author(s) declare that they have no competing interests.

## Authors' contributions

FA designed the study, implemented the computational models and wrote the manuscript. JLR and JFS selected and obtained the gene sequence data, co-interpreted the results and co-wrote the manuscript. All authors read and approved the final manuscript.
